# Survey of Obstetrician-Gynecologists about Giardiasis

**DOI:** 10.1155/2007/21261

**Published:** 2007-06-25

**Authors:** Amy Krueger, Jay Schulkin, Jeffrey L. Jones

**Affiliations:** ^1^National Center Zoonotic, Vectorborne, and Enteric Diseases (NCZVED), Coordinating Center for Infectious Diseases (CCID), Centers for Disease Control and Prevention, Mailstop F-22, 4770 Buford Highway NE, Atlanta, GA 30341-3724, USA; ^2^Department of Research, The American College of Obstetricians and Gynecologists, 409 12th Street SW, Washington, DC 20024-2188, USA

## Abstract

Giardiasis is one of the most common parasitic diseases in the United States with over 15 400 cases reported in 2005. A survey was conducted by The American College of Obstetricians and Gynecologists (ACOG) in collaboration with the Centers for Disease Control and Prevention (CDC) to evaluate the knowledge of obstetricians and gynecologists regarding the diagnosis and treatment of giardiasis. The questionnaire was distributed to a random sample of 1200 ACOG fellows during February through June of 2006. Five hundred and two (42%) responded to the survey. The respondents showed good general knowledge about diagnosis, transmission, and prevention; however, there was some uncertainty about the treatment of giardiasis and which medications are the safest to administer during the first trimester of pregnancy.

## 1. INTRODUCTION

Giardiasis is a parasitic disease caused by the protozoan parasite *Giardia intestinalis (Giardia lamblia)* [1]. *Giardia* is the most frequently reported enteric parasite in the United States and is responsible for numerous food-associated outbreaks and illnesses [2–10] as well as for waterborne disease [11–13]. Through stool examination, the prevalence of the parasite ranges from 2% to 5% in industrialized countries up to 20% to 30% in developing countries [14] largely due to a lack of adequate sanitation and hygiene [15, 16]. In the United States, giardiasis is responsible for the hospitalization of nearly 5000 people annually [17], and between 1992 and 1997 the Centers for Disease Control and Prevention (CDC) estimated that more than 2.5 million cases of giardiasis occurred annually [18]. In 2005, over 15 400 cases were reported in the United States making it the most frequently reported enteric parasitic disease [19]. The reason for the discrepancy between estimated cases and reported cases is that many persons with milder illness do not seek medical care or are not tested for *Giardia* [18].

*Giardia* can be carried by a wide variety of hosts [12, 15] and can be found in many different environments including water, soil, food, and surfaces that have been contaminated with feces from an infected human or animal [11]. The main route of exposure is fecal-oral, examples include consuming water contaminated with *Giardia*, oral contact with an item contaminated with the parasite, eating undercooked contaminated food [11, 13, 15], and in some cases oral-anal contact [15, 20, 21]. Giardiasis has long been associated with drinking contaminated water [17] or with children and workers in daycare centers [22]. The Environmental Protection Agency found *Giardia intestinalis* cysts in approximately 81% of the raw water samples collected from streams, lakes, and ponds, and in 17% of filtered water samples [23]. Fortunately most Americans do not consume raw water and are recipients of water from treatment systems that greatly decrease the chance of exposure to cysts. However, it is possible to find cysts in treated water that is inadequately filtered because of a relative resistance to chlorine [1, 24]. The prevalence of *Giardia* is as high as 35% in children attending daycare centers [14]. A previous study of children attending daycare centers in Denver, Colorado, suggests that attending a daycare center alone is a risk factor for contracting giardiasis [25].

Giardiasis can be difficult to diagnose. The illness has symptoms that are associated with a variety of parasitic, bacterial, and viral diseases; however, giardiasis should be considered when gastrointestinal symptoms last beyond several days [1]. Symptoms can include diarrhea, malaise, flatulence, greasy stools, stomach cramps, and nausea; diarrhea and malabsorption may lead to dehydration and weight loss [1, 11, 12, 26]. Another characteristic of giardiasis that can make the disease hard to identify is that cysts and trophozoites are shed on a periodic basis and stool examination may not always be performed during the time period the organism is being shed [12]. However, tests using ELISA or direct fluorescent antibody to detect antigen in the stool are more sensitive than microscopy and are now commonly available and used in the United States.

In 2005, a survey was conducted by The American College of Obstetricians and Gynecologists (ACOG) in collaboration with CDC on knowledge about common parasitic diseases [27]. The survey showed that many practitioners were not certain how to correctly prescribe medication for many of these diseases, especially which medications are safe for pregnant women [27]. The current study takes the previous survey a step further to determine knowledge about how to diagnose and treat cases of giardiasis. Malabsorption and diarrhea in pregnant women caused by giardiasis may be harmful to the fetus [28]. Along with correct diagnosis, correct treatment helps to ensure the safety of the fetus. In addition, some medications used for giardiasis may have side effects that could affect fetal development [1, 29].

## 2. METHODS

A questionnaire about two common parasitic diseases (giardiasis and toxoplasmosis) and their diagnosis and treatment by obstetrician-gynecologists was developed by ACOG and CDC and was distributed nationally by ACOG. For the purpose of this paper, we will focus on the giardiasis portion of the survey. The survey was pilot-tested by obstetrician-gynecologists in the Washington, DC, area in December 2005. ACOG mailed the survey to a random sample of 1200 out of 33 354 fellows in February 2006. To ensure the highest response rate possible, four mailing cycles were completed ending in June 2006. Data from returned surveys were assembled at the ACOG facility in Washington, DC, using SPSS [30]. Data analysis was performed at the CDC using SAS 9.1 [31]. Frequencies with confidence intervals using binomial proportions were used to convey the percentages for the survey's multiple-choice answers. The mean ages of the total population and survey sample were compared with the *Z* test; other demographic variable proportions were compared with the chi-square. The survey was reviewed and exempted by human subjects staff at ACOG and CDC.

## 3. RESULTS

Of the 1200 ACOG fellows who were mailed the survey, 502 responded for a response rate of 42%. Table 1 displays the demographics for the participants including gender, location, and type of practice, as well as statistical differences between the survey population and the ACOG member population. The survey population had a slightly lower mean age than the ACOG member population (46 years versus 47 years, resp., *P* = .001). 

Generally, the participants answered the survey questions correctly, although for a few questions there was a lot of uncertainty. Medication used for the treatment of giardiasis was one area where fewer of the participants indicated the most correct answer. Approximately half (49.6%) of the participants chose metronidazole, which is used for treatment of giardiasis; however many participants did not recognize that mebendazole is not a primary treatment of giardiasis, and that tinidazole and nitazoxanide can also be used for treatment. The participants also did not usually select the safest medication to use for pregnant women in the first trimester. The majority (75.8%) believed metronidazole to be the safest, while in actuality paromomycin is the safest treatment in the first trimester (although less effective). The practitioners (66.8%) also believed that the treatment of asymptomatic carriers is recommended, however it is not the recommended practice in most cases. Table 2 shows the distributions for each survey question.

## 4. DISCUSSION

The objective of this study was to create a concise survey that would cover the basics in knowledge about giardiasis. Through this survey, we found that the majority of obstetrician-gynecologists provided correct information regarding giardiasis; however, the survey also showed areas where further education is needed. Most physicians correctly answered questions about how the disease is transmitted, prevention methods, and outcomes of the disease. However, one of the most important issues concerning the disease is treatment and many of the participants might benefit from further education in this area (Table 2). 

## 5. MEDICATIONS USED FOR TREATMENT OF GIARDIASIS

Gardner and Hill [1] provide a thorough review of drugs for treatment of giardiasis including medications for the use in pregnant women. The largest class of agents to treat *Giardia* is the nitroimidazoles, which includes metronidazole and tinidazole. Metronidazole is the most common drug used to treat giardiasis worldwide [1]. It has been found to have an efficacy of 85%–90% in adult and pediatric patients [1, 32]. Tinidazole is one of the drugs with potential for the greatest compliance since it has a longer half-life and can be taken in one dose [1, 33, 34]. In 2004, tinidazole was approved for use in the United States [35]. Studies have shown the drug to have a median efficacy of 92%, and up to 100% for a one-dose regimen [1, 36]. Nitazoxanide was approved for treatment of *Giardia* in the US in 2003 [16]. A study in Mexico found nitazoxanide to have an efficacy rate of 56%–74%, while other studies have found efficacy rates as high as 80% [16]. An in vitro study showed that nitazoxanide is more potent than albendazole and metronidazole, 2.5 and 50 times, respectively [37]. Clinical trials with mebendazole have given varying results, and thus other therapies are preferentially recommended [1, 38–41]. Trials comparing mebendazole to metronidazole showed that mebendazole was less effective against giardial infections [38–40]. In the survey, the best answer was to recognize that mebendazole is not the preferred method of treatment (in Table 2 “all except mebendazole” was chosen by 45.7%). However, many participants (49.6%) chose metronidazole as the medication to use for treatment, which is correct, as well as tinidazole (chosen by 0.2%). Therefore, 95.6% of participants indicated at least one of the correct medications for treatment of giardiasis.

## 6. SAFEST MEDICATIONS TO USE DURING FIRST TRIMESTER OF PREGNANCY

Metronidazole has been found to be carcinogenic in rats and mice but has not been proven so in humans [1, 32]. Metronidazole rapidly enters the fetal circulation after absorption by the mother, which raises concerns about the use during pregnancy [1]. Some studies have shown no harmful effects to the fetus [42], and the drug falls in FDA pregnancy category B for teratogenic effects [27]. Many studies have found metronidazole to be safe for treatment during the second and third trimesters [1, 32, 42]. Over 75% of the participants believed metronidazole to be the safest medication for use in the first trimester. Since tinidazole is in the same family as metronidazole, it displays similar side effects [1]. A case-control study of oral tinidazole treatment has shown placental transfer; it is not generally recommended for use in the first trimester of pregnancy much like its relative, metronidazole [43]. Of the respondents in our survey 6.7% considered tinidazole to be the safest treatment in the first trimester.

Albendazole is in the same family as mebendazole and has also shown inconsistencies in its effectiveness when used alone [1]. Albendazole has been found to be teratogenic in mice and rats and is not generally recommended for use in pregnant women, especially during the first trimester [1, 29]. In the current survey, 1.3% of the participants considered albendazole to be the safest medication for treatment in pregnant women. Paromomycin is considered the safest to use for treatment in the first trimester because it is poorly absorbed from the intestine and nearly 100% is excreted unchanged; therefore, little if any of the drug reaches the fetus [1, 36]. In addition, no teratogenicity has been found with this treatment [36]. Paromomycin has been found to have an efficacy of 55%–90% [1]. Approximately 16% of respondents indicated that paramomycin was the safest medication to use in the first trimester of pregnancy. Although paromomycin is theoretically the safest treatment in the first trimester, it is not necessarily the least expensive or most available. 

## 7. TREATMENT OF ASYMPTOMATIC CARRIERS

Screening and treatment of asymptomatic carriers is not generally recommended but depends on the specific situation in which the patient resides. It is often not desirable to treat asymptomatic persons because people often become recolonized [1], and even with intensive investigation and treatment in daycare centers outbreaks can recur [44]. However, it may be necessary to treat if the disease contributes to underdevelopment in children. In the United States, most children have good nutritional status and in turn may not have any adverse health effects from colonization; however, treatment should be considered if spread of the disease is likely, for example, in a household when it has spread from person to person [1]. Resistance is another consideration for not treating asymptomatic carriers. The overuse of a drug may cause resistance [1] which could affect treatment courses in the future. 

## 8. LIMITATIONS

One limitation of the survey results is the low response rate (42%). Respondents who are more knowledgeable about giardiasis may have been more likely to complete the questionnaire, leading to an overestimate of knowledge. Nevertheless, the study population was similar to the overall ACOG membership and we were able to identify subject areas where continuing education would be beneficial. Another limitation is the lack of a fixed denominator. For some questionnaires, not all participants completed all the questions. This could also affect the estimate of the knowledge of the physicians.

## 9. EDUCATION

Through the survey, we found that over 41% of obstetrician-gynecologists use journals as their main source of new information. Approximately, half of the participants also expressed interest in featured articles by ACOG as a way to impart educational material. Results from our survey will be used to inform ACOG fellows through reports such as this one.

## Figures and Tables

**Table 1:** Demographics for the survey sample of obstetrician-gynecologists and the American College of Obstetricians and Gynecologists (ACOG), 2006.

Characteristic		Number	Study (*N* = 502) (%)	ACOG members (*N* = 33 354) (%)	Population comparison *P*-value

Gender (*N* = 502)	Male	254	50.6	51.8	0.9056
	Female	248	49.4	48.2	

Age (mean; years) (*N* = 500)		500	46	47	0.001

ACOG districts* (*N* = 468)	District 1	27	5.8	6.2	0.9081
	District 2	14	3.0	6.5	
	District 3	50	10.7	7.3	
	District 4	77	16.4	20.6	
	District 5	63	13.5	10.3	
	District 6	34	7.3	10.2	
	District 7	105	22.4	19.3	
	District 8	48	10.3	10.2	
	District 9	50	10.7	9.0	

Practice type (*N* = 502)	Solo	95	18.9	21.5	0.6825
	Military	11	2.2	5.8	
	OB/GYN partnership/group	286	57.0	48.9	
	University	61	12.2	13.4	
	Other including HMO	49	9.8	10.5	

*US states and territories in districts are defined as follows: District 1: Connecticut, Maine, Massachusetts, New Hampshire, Rhode Island, Vermont; District 2: New York; District 3: Delaware, New Jersey, Pennsylvania; District 4: District of Columbia, Florida, Georgia, Maryland, North Carolina, South Carolina, Virginia, West Virginia, Puerto Rico, US West Indies; District 5: Indiana, Kentucky, Ohio, Michigan; District 6: Illinois, Iowa, Minnesota, Nebraska, North Dakota, South Dakota, Wisconsin; District 7: Alabama, Arkansas, Kansas, Louisiana, Mississippi, Missouri, Oklahoma, Tennessee, Texas; District 8: Alaska, Arizona, Colorado, Hawaii, Idaho, Montana, Nevada, New Mexico, Oregon, Utah, Washington, Wyoming, American Samoa; District 9: California.

**Table 2:** Frequencies and confidence intervals for giardiasis survey questions, sample of obstetrician-gynecologists in the American College of Obstetricians and Gynecologists, 2006.

Quastion		Number	(%)	95% confidence interval (%)

Symptoms of giardiasis (choose two): *N* = 988 (all choices)	*Abdominal cramps	468	47.4	44.3, 50.5
	*Diarrhea	440	44.5	41.4, 47.6
	Edema	0	0.0	—
	Blood diarrhea	80	8.1	6.4, 9.8

Can lead to malabsorption: *N* = 493	*True	454	92.1	89.3, 94.3
	False	39	7.9	5.5, 10.3

Contracted from: *N* = 477	Drinking untreated water	159	33.3	29.1, 37.8
	Swallowing water while swimming in pools	0	0.0	—
	Another person, toddlers, children in daycare	4	0.8	0.2, 2.1
	Contaminated food	7	1.5	0.6, 3–3.0
	*All of the above	307	64.4	60.1, 68.7

Classic method for diagnosis (microscopy) may miss the organism (even after 3 stool specimens): *N* = 497	*True	453	91.1	88.3, 93.5
	False	44	8.9	6.4, 11.4

Laboratory diagnosis is often made by which test: *N* = 493	Blood smear	8	1.6	0.5, 2.7
	Serum antibody test	45	9.1	6.59, 11.7
	*Enzyme immunoassay (to detect antigens in stool)	440	89.2	86.5, 92–72.0

Most common reason pregnant women with giardiasis are hospitalized: *N* = 498	Anemia	11	2.2	0.9, 3.5
	*Dehydration	480	97.8	94.8, 98–98.0
	Amniocentesis	0	0.0	—
	Premature rupture of membranes	7	1.4	0.4, 2.4

Medications used for giardiasis: *N* = 472	Tinidazole	1	0.2	0.0, 0.6
	Metronidazole	234	49.6	45.1, 54.1
	Nitazoxanide	0	0.0	—
	Mebendazole	21	4.4	2.6, 6.3
	*all except Mebendazole	216	45.7	41.3, 50.3

Safest medication to use in first trimester: *N * = 463	Tinidazole	31	6.7	4.4, 9–4.0
	Metronidazole	351	75.8	71.9, 79.7
	Albendazole	6	1.3	0.3, 2.3
	*Paromomycin	75	16.2	12.8, 19.6

Should wait two weeks after giardiasis before swimming: *N* = 481	*True	295	61.3	56.8, 65.7
	False	186	38.7	34.3, 43–43.0

Most common enteric parasitic disease in US: *N* = 488	*True	387	79.3	76.1, 83.1
	False	99	20.3	16.8, 24–24.0

Toddlers, mothers, and care takers most at risk: *N* = 489	*True	384	78.5	74.9, 82.2
	False	105	21.5	17.8, 25.1

Incubation period is 1–4 weeks: *N* = 489	*True	434	88.8	85.6, 91.4
	False	55	11.2	8.5, 14.1

Treatment of asymptomatic carriers is not usually recommended: *N* = 491	*True	163	33.2	29.0, 37.4
	False	328	66.8	62.6, 71–71.0

*Correct answer
